# The Role of Preoperative Neutrophil-to-Lymphocyte Ratio and C-reactive Protein in Predicting the Severity of Cholelithiasis With Cholecystitis

**DOI:** 10.7759/cureus.105636

**Published:** 2026-03-22

**Authors:** Kanishk K, Shivanagouda S Patil, Ramakanth Baloorkar, Shailesh Kannur, Mruthunjaya S Bevinamarad, Nagaraj Biradar, Bhuvaneshwari Gachinmath

**Affiliations:** 1 General Surgery, Shri B.M. Patil Medical College, Hospital and Research Centre, BLDE (Deemed to be University), Vijayapura, IND

**Keywords:** acute cholecystitis, cholelithiasis, c-reactive protein, disease severity, neutrophil-to-lymphocyte ratio

## Abstract

Introduction: Acute calculous cholecystitis is a common surgical condition with variable clinical severity. Early identification of severe disease remains challenging. Inflammatory biomarkers such as neutrophil-to-lymphocyte ratio (NLR) and C-reactive protein (CRP) have been proposed as simple indicators of systemic inflammation and disease severity. The present study aimed to evaluate the association of preoperative NLR and CRP levels with the severity of calculous cholecystitis and their relationship with clinical outcomes.

Materials and methods: This prospective observational study was conducted over two years in a tertiary care centre and included 48 patients diagnosed with cholelithiasis with cholecystitis and undergoing laparoscopic cholecystectomy. Preoperative NLR and CRP levels were recorded within 24 hours prior to surgery. Based on intraoperative findings and histopathology, patients were classified into simple, purulent, and gangrenous cholecystitis. Correlation analysis was performed to assess the relationship between inflammatory markers and duration of hospital stay.

Results: The mean age was 45.08 ± 13.78 years, with female predominance 30 (62.5%). Simple cholecystitis was most common 23 (47.9%), followed by purulent 15 (31.3%) and gangrenous 10 (20.8%). Median NLR and CRP were 2.67 and 10.55 mg/dl, respectively. Both NLR and CRP increased significantly with worsening severity (p < 0.001). NLR showed a strong positive correlation with CRP (r = 0.641, p = 0.001). NLR (r = 0.427, p = 0.001) and CRP (r = 0.539, p = 0.001) correlated significantly with longer hospital stay. Age and BMI were not significantly associated with severity.

Conclusion: Preoperative NLR and CRP levels are significantly associated with the severity of calculous cholecystitis and duration of hospitalization. These readily available biomarkers may serve as useful adjuncts in clinical severity assessment.

## Introduction

Acute cholecystitis is a common condition encountered in hepatobiliary surgical practice and most frequently results from obstruction of the cystic duct by gallstones, accounting for nearly 90% of cases [1]. The prevalence of gallstones in the Asian population ranges from 3% to 5% [2]. Cholelithiasis occurs more commonly in women, particularly during pregnancy, in elderly individuals, and in patients with dyslipidaemia [3]. Persistent cystic duct obstruction leads to gallbladder distension, inflammation, and in severe cases, ischemia and necrosis [4].

Clinically, patients present with right upper quadrant pain, positive Murphy’s sign, fever, vomiting, and occasionally radiation of pain to the right subscapular region [5]. Elderly patients may exhibit atypical or less pronounced symptoms, leading to delayed presentation [6]. If untreated, acute cholecystitis may progress to gangrene, perforation, peritonitis, sepsis, and shock [7]. Severe forms have been reported in 22-30% of cases; however, early recognition remains challenging due to variability in clinical features and imaging findings and the lack of consistently applied standardized severity grading systems in routine clinical practice [1,8].

Inflammatory biomarkers such as the neutrophil-to-lymphocyte ratio (NLR) and C-reactive protein (CRP) have been increasingly investigated as indicators of systemic inflammation [9]. Neutrophils contribute to innate immune defence through chemotaxis, phagocytosis, and cytokine release, whereas lymphocytes represent adaptive immune responses [10]. The NLR reflects the balance between these two components and has been proposed as a marker of inflammatory burden [11]. CRP, a homopentameric acute-phase protein synthesized primarily by the liver, rises in response to inflammatory stimuli and is widely used as a marker of acute inflammation [12].

Although imaging modalities such as ultrasonography, CT, and MRI are routinely used to assess cholecystitis severity, their ability to consistently stratify disease severity remains limited [13]. Therefore, the present study aimed to evaluate the association of preoperative NLR and CRP levels with the severity of calculous cholecystitis using intraoperative and histopathological findings as practical indicators of disease severity and their relationship with clinical outcomes.

## Materials and methods

This prospective observational study was conducted in the Department of General Surgery at Shri B.M. Patil Medical College, Hospital and Research Centre, BLDE (DU) between June 2024 and December 2025. The study included patients diagnosed with cholelithiasis with cholecystitis and scheduled for laparoscopic cholecystectomy. Ethical approval was obtained from the Institutional Ethics Committee (IEC No.: BLDE(DU)/IEC-SBMPMC/093/2023-24), and written informed consent was obtained from all participants prior to enrolment.

The sample size was calculated using the formula,

n = (Z x σ / d)^2^

based on a previously reported mean NLR of 10.2 with SD 3.85, considering a 99% confidence level (Z = 2.58), margin of error of 1.5, and alpha level of 0.01, yielding a minimum required sample of 48 patients [1].

Patients diagnosed with cholelithiasis and cholecystitis on ultrasonography, abdominal CT, or MRI and planned for laparoscopic cholecystectomy were included in the study. Patients with gallbladder carcinoma, cholangiocarcinoma, acalculous cholecystitis, immunocompromised status, those on hormone replacement therapy, and pregnant women were excluded.

For each patient, demographic details, clinical history, and comorbidity profile were recorded using a pretested structured proforma. Preoperative laboratory investigations included complete blood count with differential leukocyte count, calculation of NLR, CRP, and liver function tests performed within 24 hours prior to surgery. NLR was calculated by dividing the absolute neutrophil count by the absolute lymphocyte count. Intraoperative findings were used to classify cholecystitis into simple, purulent, or gangrenous types based on macroscopic appearance of the gallbladder, presence of pus, necrosis, or gangrenous changes [1,14]. Postoperative outcomes, including duration of hospital stay and need for conversion to open surgery, were recorded. Patients were discharged based on clinical stability, including resolution of symptoms, ability to tolerate oral intake, and absence of postoperative complications. Histopathological examination of the resected gallbladder specimen was reviewed to confirm the diagnosis.

Data were entered into Microsoft Excel and analyzed using Statistical Package for the Social Sciences (SPSS) v26. Continuous variables were expressed as mean ± standard deviation or median with interquartile range as appropriate. Categorical variables were presented as frequency and percentage. Pearson’s correlation was used to assess the relationship between inflammatory markers and duration of hospital stay. One-way analysis of variance (ANOVA) was applied to compare laboratory parameters across different types of cholecystitis. Multivariate analysis was not performed due to the limited sample size. A p-value < 0.05 was considered statistically significant.

## Results

The study included 48 patients with a mean age of 45.08 ± 13.78 years, most commonly in the 36-50 years group (22 (45.8%)). Females predominated 30 (62.5%). The mean BMI was 25.12 ± 1.98 kg/m². Diabetes mellitus was the most frequent comorbidity 8 (16.7%), followed by hypertension 3(6.3%) (Table 1).

**Table 1:** Baseline demographic characteristics and comorbidities of the study population (n = 48)

Variable	Category	n (%) / Mean ± SD
Age (years)	20–35 years	13 (27.1%)
	36–50 years	22 (45.8%)
	51–80 years	13 (27.1%)
	Mean ± SD	45.08 ± 13.78
Gender	Female	30 (62.5%)
	Male	18 (37.5%)
BMI (kg/m²)	Mean ± SD	25.12 ± 1.98
Comorbidities	Diabetes mellitus	8 (16.7%)
	Hypertension	3 (6.3%)
	Ischemic heart disease	2 (4.2%)
	Chronic liver disease	1 (2.1%)

Baseline hematological evaluation showed leucocytosis with a mean white blood cell count (WBC) of 9994.5 ± 4,575.1/mm³ and neutrophil predominance (66.44 ± 11.59%). Liver enzymes were mildly elevated overall, with mean aspartate aminotransferase (AST) 37.27 ± 28.31 U/L and alanine aminotransferase (ALT) 28.1 ± 9.55 U/L (Table 2).

**Table 2:** Hematological and liver function tests Baseline hematological parameters and liver function test profile of the study participants. WBC: White blood cell count; AST: Aspartate aminotransferase; ALT: Alanine aminotransferase; ALP: Alkaline phosphatase

Parameter	Mean ± SD	Range of values observed in present study
Hemoglobin (g/dL)	12.27 ± 1.83	7.4-17.5
WBC (/mm³)	9994.5 ± 4575.1	1480-28110
Neutrophils (%)	66.44 ± 11.59	46.6-91.5
Lymphocytes (%)	25.53 ± 9.97	4-46.6
Total Bilirubin (mg/dL)	0.93 ± 0.35	0.27-2.00
AST (U/L)	37.27 ± 28.31	12-140
ALT (U/L)	28.1 ± 9.55	12-58
ALP (U/L)	93.6 ± 26.09	46-175

The median NLR was 2.67 (interquartile range (IQR) 2.49), while median CRP was 10.55 (IQR 23.2), indicating variability in inflammatory response among the study population (Table 3).

**Table 3:** NLR and CRP among patients Distribution of inflammatory markers - NLR and CRP - expressed as median with 95% confidence interval and IQR. NLR: Neutrophil-to-lymphocyte ratio; CRP: C-reactive protein; IQR: Interquartile range

	Median	95% confidence interval		IQR
		Lower bound	Upper bound	
NLR	2.67	2.53	4.48	2.49
CRP (mg/dl)	10.55	17.88	36.44	23.2

Simple cholecystitis was most common 23 (47.9%), followed by purulent 15 (31.3%) and gangrenous 10 (20.8%). Conversion to open surgery occurred in 2 (4.2%) cases. The mean hospital stay was 5.47 ± 1.72 days (Table 4).

**Table 4:** Clinical characteristics of cholecystitis and surgical outcomes among the study participants

Variable	Category	n (%) / Mean ± SD
Type of cholecystitis	Simple	23 (47.9%)
	Purulent	15 (31.3%)
	Gangrenous	10 (20.8%)
Conversion to open surgery	Yes	2 (4.2%)
Duration of hospital stay (days)	Mean ± SD	5.47 ± 1.72

Significant positive correlations were observed between NLR and CRP (r = 0.641, p = 0.001). Both NLR (r = 0.427, p = 0.001) and CRP (r = 0.539, p = 0.001) correlated with longer hospital stay, suggesting an association between elevated inflammatory markers and increased disease burden (Table 5).

**Table 5:** Correlation of inflammatory markers with hospital stay Correlation analysis of inflammatory markers (NLR and CRP) with duration of hospital stay using Pearson’s correlation coefficient. p value < 0.05 was considered statistically significant. NLR: Neutrophil-to-lymphocyte ratio; CRP: C-reactive protein

Variables compared	Pearson’s r	p-value
NLR vs CRP	0.641	0.001
NLR vs hospital stay	0.427	0.001
CRP vs hospital stay	0.539	0.001

On subgroup analysis, NLR and CRP increased significantly with worsening cholecystitis severity (p < 0.001). Neutrophil percentage rose while lymphocyte percentage declined significantly across groups (p < 0.001). Hospital stay was significantly longer in gangrenous and purulent cases compared to simple cholecystitis (p < 0.001). Age and BMI showed no significant difference across severity groups (Table 6).

**Table 6:** Comparison of clinical and laboratory parameters by type of cholecystitis Comparison of clinical and laboratory parameters among different types of cholecystitis using one-way ANOVA. p value < 0.05 was considered statistically significant. NLR: Neutrophil-to-lymphocyte ratio; CRP: C-reactive protein; ANOVA: Analysis of variance

Parameter	Gangrenous (n=10)	Purulent (n=15)	Simple (n=23)	F-value	p-value
Age (years)	42.5 ± 13.9	43.4 ± 16.5	47.3 ± 12.0	0.53	0.59
NLR	7.33 ± 5.84	3.54 ± 0.76	1.82 ± 0.59	19.84	<0.001
CRP (mg/dl)	83.8 ± 19.5	20.8 ± 12.1	6.7 ± 2.7	128.72	<0.001
Neutrophils (%)	79.76 ± 7.36	69.94 ± 9.45	58.8 ± 7.42	21.46	<0.001
Lymphocytes (%)	14.88 ± 7.32	20.42 ± 4.23	33.51 ± 6.57	33.28	<0.001
BMI (kg/m²)	26.48 ± 1.94	24.94 ± 2.43	24.66 ± 1.42	2.65	0.081
Hospital stay (days)	6.8 ± 1.4	6.1 ± 1.9	4.5 ± 1.0	12.91	<0.001

## Discussion

Inflammatory biomarkers such as NLR and CRP have been increasingly evaluated as indicators of systemic inflammatory response in acute cholecystitis. Neutrophils reflect innate immune activation, whereas lymphocytes represent adaptive immunity, and the NLR integrates these two pathways [10]. CRP, an acute-phase reactant synthesized by hepatocytes, rises significantly during inflammatory states [12]. In the present study, both NLR and CRP showed significant positive correlation with duration of hospital stay, and NLR demonstrated a strong positive correlation with CRP levels. These findings suggest that increasing inflammatory burden parallels greater clinical severity and resource utilization.

The demographic profile in this study demonstrated a mean age of 45.08 ± 13.7 years with female predominance 30 (62.5%), consistent with previously reported epidemiological trends. Saqib et al. reported a mean age of 35.81 ± 8.12 years with 49 (71%) female patients [2]. Similarly, Mahmood et al. observed 118 (67%) female patients with a median age of 51 years [9]. These comparable findings reinforce the established female preponderance and middle-aged predominance in gallstone-related cholecystitis.

In the present study, inflammatory markers varied significantly with disease severity. Patients with gangrenous cholecystitis had markedly higher NLR and CRP values compared to purulent and simple cholecystitis. Neutrophils increased, while lymphocytes declined significantly with increasing severity. However, age and BMI were not significantly associated with disease severity. Similar observations were reported by Xia et al., who demonstrated significantly higher preoperative NLR and CRP values in purulent and gangrenous cholecystitis (p < 0.05) [1]. Beliaey et al. showed that NLR had high diagnostic accuracy with an area under the curve (AUC) of 0.94 and was superior to total leukocyte count in identifying acute cholecystitis [15]. Uludağ et al. also reported significantly elevated WBC, CRP, and NLR levels in complicated cholecystitis [16]. Mahmood et al. identified age, CRP, and NLR as independent predictors of severity [9]. While previous studies have explored predictive roles of these biomarkers, the present study design allows only assessment of association, and no causal or independent predictive inference can be established.

With respect to clinical outcomes, simple cholecystitis was the most common presentation, and laparoscopic cholecystectomy was successfully completed in the majority of cases with a low conversion rate of 2 (4.2%). The mean hospital stay was 5.47 ± 1.72 days, with significantly longer stay observed in gangrenous and purulent cholecystitis. Mahmood et al. reported a median hospital stay of four days with a 4 (2%) conversion rate and low postoperative complication rates [9]. The observed association between elevated inflammatory markers and prolonged hospitalization in the present study supports their utility as markers of disease burden and severity in acute calculous cholecystitis.

Strengths and limitations

The present study has several strengths, including its prospective design, uniform preoperative laboratory evaluation, and objective intraoperative classification of cholecystitis severity, which enhanced data consistency and reduced recall bias. The simultaneous assessment of NLR and CRP and their correlation with clinical outcomes such as hospital stay adds practical clinical relevance. However, the study has certain limitations, notably the relatively small sample size from a single center, which may limit generalizability. Additionally, multivariate analysis was not performed to establish independent predictive value, and long-term postoperative outcomes were not assessed. Furthermore, standardized severity grading systems, such as the Tokyo Guidelines 2018, were not applied, and severity classification was based on intraoperative findings; this may have limited comparability with other studies and standardized clinical stratification.

## Conclusions

The present study demonstrates that elevated preoperative NLR and CRP levels are significantly associated with increasing severity of calculous cholecystitis and prolonged hospital stay. Patients with gangrenous and purulent cholecystitis exhibited markedly higher inflammatory markers compared to those with simple disease. Although these markers showed strong association with disease severity, their independent predictive value requires further validation in larger studies using standardized severity grading systems. NLR and CRP, being simple and readily available laboratory parameters, may serve as useful adjuncts in the clinical assessment and risk stratification of patients with acute calculous cholecystitis; however, their role as definitive predictors of severity requires further investigation.
